# MHSNMF: multi-view hessian regularization based symmetric nonnegative matrix factorization for microbiome data analysis

**DOI:** 10.1186/s12859-020-03555-w

**Published:** 2020-11-18

**Authors:** Yuanyuan Ma, Junmin Zhao, Yingjun Ma

**Affiliations:** 1 School of Computer & Information Engineering, Anyang Normal University, Anyang, China; 2 School of Computer & Data Science, Henan University of Urban Construction, Pingdingshan, China; 3 School of Computer, Central China Normal, Wuhan, China

**Keywords:** Symmetric nonnegative matrix factorization, Hessian regularization, Multi-view clustering, Human microbiome

## Abstract

**Background:**

With the rapid development of high-throughput technique, multiple heterogeneous omics data have been accumulated vastly (e.g., genomics, proteomics and metabolomics data). Integrating information from multiple sources or views is challenging to obtain a profound insight into the complicated relations among micro-organisms, nutrients and host environment. In this paper we propose a multi-view Hessian regularization based symmetric nonnegative matrix factorization algorithm (MHSNMF) for clustering heterogeneous microbiome data. Compared with many existing approaches, the advantages of MHSNMF lie in: (1) MHSNMF combines multiple Hessian regularization to leverage the high-order information from the same cohort of instances with multiple representations; (2) MHSNMF utilities the advantages of SNMF and naturally handles the complex relationship among microbiome samples; (3) uses the consensus matrix obtained by MHSNMF, we also design a novel approach to predict the classification of new microbiome samples.

**Results:**

We conduct extensive experiments on two real-word datasets (Three-source dataset and Human Microbiome Plan dataset), the experimental results show that the proposed MHSNMF algorithm outperforms other baseline and state-of-the-art methods. Compared with other methods, MHSNMF achieves the best performance (accuracy: 95.28%, normalized mutual information: 91.79%) on microbiome data. It suggests the potential application of MHSNMF in microbiome data analysis.

**Conclusions:**

Results show that the proposed MHSNMF algorithm can effectively combine the phylogenetic, transporter, and metabolic profiles into a unified paradigm to analyze the relationships among different microbiome samples. Furthermore, the proposed prediction method based on MHSNMF has been shown to be effective in judging the types of new microbiome samples.

## Background

With the rapid development of bio-technique, such as high-through sequencing technique, plenty of multiple omics data (e.g. metagenomics, metabolomics and so on) have generated in microbiome study. These resources pave the way for researchers to explore and understand the structure and functions of microbiome community. In addition, it helps to reveal the relationships between microbiota and host environment, microbes and diseases. In order to further dissect the structure and functions of microbiome, many microbiome projects including Human Microbiome Plan (HMP) [1], Integrative Human Microbiome Plan (iHMP) [2], and Metagenomics of the Human Intestinal Gut (MetaHIT) [3] have been launched and accumulated large amounts of microbiome data. By some analysis tools, these data can be computationally represented as the phylogenetic profile or functional composition profile of microbiome [4]. Although some approaches have been designed to analyze the difference and connections among different microbiome samples, they only considered one kind of biological profile data. Thus, the conclusions obtained from these approaches may be one-sided and incorrect. In order to draw a reasonable conclusion, integrating multiple omics data from different biological scenarios to jointly analyze latent patterns becomes a feasible way.

However, to the best of our knowledge, there have been few approaches to simultaneously combine multiple biological profiles into a paradigm to study the underlying microbiome structure shared by different representations. Hence, it is urgent and necessary to design novel data integration methods or tools to explore the complicated relationship among microorganisms.

As a kind of clustering method, nonnegative matrix factorization (NMF) has drawn great public attention, recently. In text mining, image processing, bioinformatics fields and so on, many new data integration methods based on NMF have emerged. Greene et.al proposed a joint nonnegative matrix factorization algorithm by concatenating the features of all the views to form a new representation, and then it was factorized into two low rank matrices, one of which was used to cluster indicator [5]. Liu et.al proposed the Multi-NMF algorithm by searching a common consensus solution across different views [6]. Zhang et.al developed a novel NMF framework (CSMF) to reveal the common and specific patterns obtained from multiple interrelated biological scenarios [7]. All these methods could obtain good performance when data distribution satisfies certain conditions, e.g. linear relationship. However, the real-world data often owns complicated structure and nonlinear relation. For example, the interactions among microbes are easily influenced by the food intake, host environment or other species, particularly for the intestinal flora, and thus the relationship among microbes may be delicate and complicated. Traditional approaches based on NMF are not sufficient for revealing the latent relations hidden in multiple biological data profiles.

In order to improve the clustering performance, Laplacian graph which makes use of the geometric information of the original data was introduced into the NMF framework. Cai et.al proposed a graph regularization based nonnegative matrix factorization approach (GNMF) for data clustering and obtained good performance [8]. Jiang et.al proposed a new joint nonnegative matrix factorization algorithm with robust Laplacian graph (LJ-NMF) to cluster microbiome data [4] and achieved better clustering performance. Chen et.al proposed a novel co-module mining framework based on Tri-factor nonnegative matrix factorization (NetNMF) to identify heterogeneous biological modules [9] and easily extended to Laplacian case with prior knowledge. Even though Laplacian can boost the performance, Kim et.al pointed that Laplacian regularization possibly leaded poor extrapolating power because Laplacian regularization always biased the solution towards a constant function [10]. Compared to Laplacian regularization, Hessian can not only effectively exploit the local geometry information of original data, but also extrapolate beyond data points [11].

To solve the above problems, in this paper we propose a novel multi-view Hessian regularization based symmetric nonnegative matrix factorization algorithm (MHSNMF) to integrate multiple biological profiles into an unified framework to analyze the potential clustering patterns across all view. MHSNMF utilizes the local geometrical information of different views and automatically assigns corresponding weights for each view in each iteration process. We conduct large amounts of experiments on two real datasets and the experimental results show that the proposed MHSNMF algorithm outperforms other integrating approaches, suggesting its underlying application in microbiome data analysis.

**Fig. 1:**
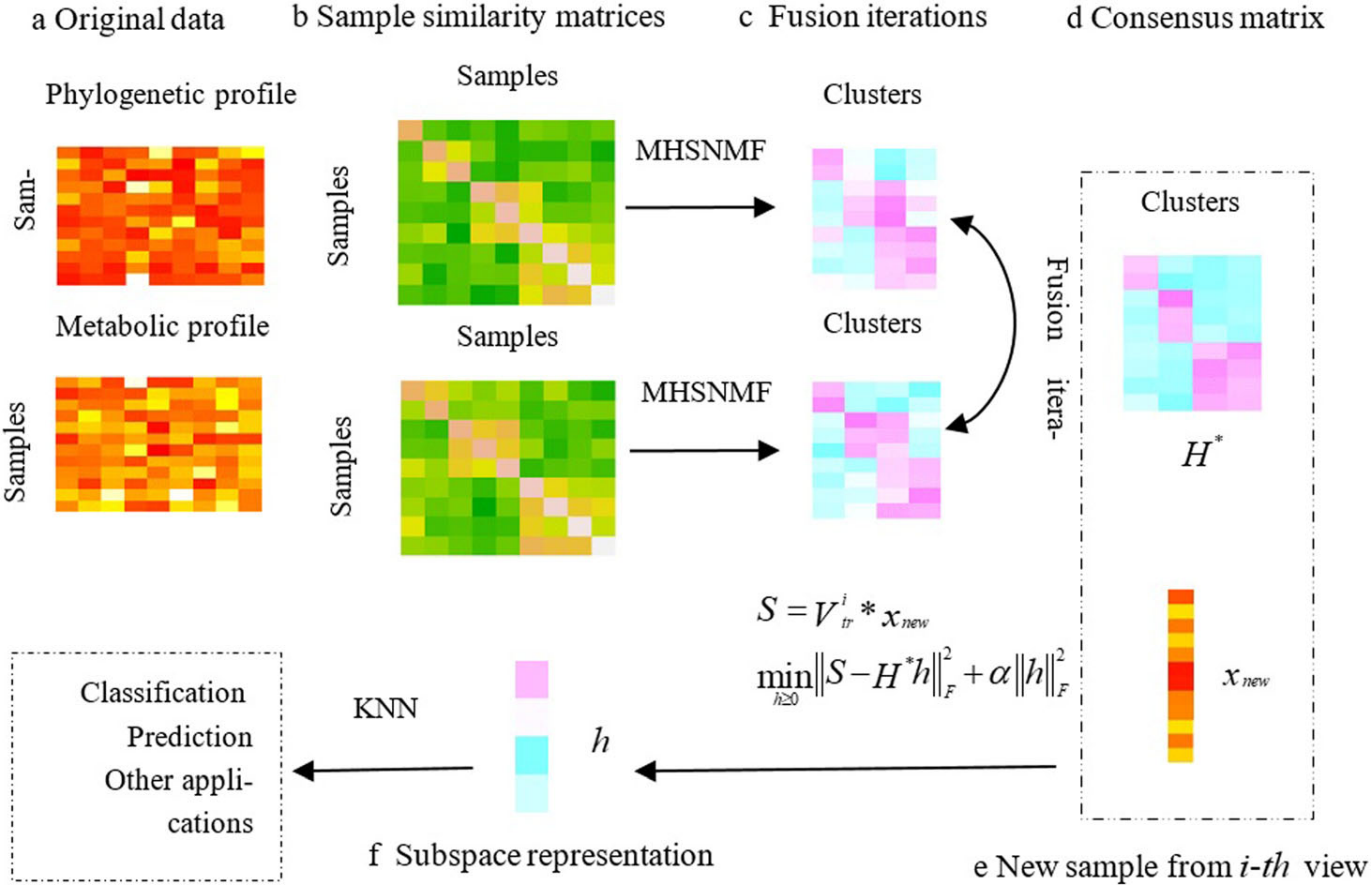
Illustrative of MHSNMF framework on human microbiome data. **a** Example representation of the phylogenetic profile and metabolic profile for the same cohort of samples. **b** Sample-sample similarity matrices obtained from each view. **c** Using MHSNMF, each similarity matrix is factorized into a low rank matrix and its transposition. Matrix fusion process iteratively updates each clustering with information from the other view. **d** The iterative fusion leads to convergence to the final consensus matrix *H*^∗^. **e** Given a new sample *x*_*new*_ from the *i* ‐ *th* view, we can obtain its subspace representation *h* by *H*^∗^ and the proposed mapping approach. Here, ${V}_{tr}^i$ indicates the training samples from *i* ‐ *th* view, *S* denotes the similarity between *x*_*new*_ and ${V}_{tr}^i$. *α* is the regularization parameter. **f** Once obtaining *h*, some applications such as classification, prediction and so on would be executed naturally

The contributions of this study lie in: (1) an effective integration method to explore the difference among distinct microbiome samples with multiple views has been proposed. The experimental results show that it outperforms the state-of-art algorithms in terms of AC and NMI; (2) high-order information of the original data is exploited to reveal the underlying clustering patterns across different views; (3) a novel approach based on the consensus matrix obtained from MHSNMF is proposed to predict the classification of new microbiome samples. The extended experiments demonstrate the effectiveness of the proposed method. Figure 1 demonstrates the flowchart of MHSNMF algorithm.

The rest of this paper is organized as below: in next section, a brief view of SNMF and multi-view clustering is provided, and then multi-view Hessian regularization based SNMF algorithm is also proposed. Next extensive experiments results and the comparisons with other methods are presented. At last, the conclusion and next research plans are given.

## Methods

### Symmetric nonnegative matrix factorization

Nonnegative matrix factorization (NMF), which has been widely used in many fields including text clustering, image recognition, bioinformatics, has drawn great attention. In NMF, the data matrix *V* is factorized the production of two low rank matrices *W* and *H*. Each column *V*_.*i*_ in original matrix *V* can be approximated as the linear combination of basis vectors *W*_.*j*_, the coefficients are the corresponding elements of *H*_.*i*_. Hence, when data owns linear structure, NMF can achieve better performance. However, the real world data distribution is usually complex and hard to dissect the relations among different objects, and especially for the microbial data. Symmetric nonnegative matrix factorization (SNMF) views the data samples as vertices in graph and minimizes certain objective function of graph cuts [12]. SNMF can adopt multiple metrics to character the similarities between two nodes, including inner kernel, Gaussian kernel, correlation coefficient methods and so on.

The objective function of SNMF is defined as:

1 $$ O=\underset{H\ge 0}{\mathit{\operatorname{Min}}}{\left\Vert A-H{H}^T\right\Vert}_F^2. $$

where ‖∗‖_*F*_ is the Frobenius norm of matrix, $A\in {R}_{+}^{n\times n}$ is the similarity matrix, and $H\in {R}_{+}^{n\times k}$ is the factorized low-rank matrix, *k* is the degree of factorization. *A*_*ij*_ denotes the similarity between *i* ‐ *th* and *j* ‐ *th* node.

Eq. 1 iteratively updates *H* using the following rule [11, 13]:

2 $$ {H}_{ij}\leftarrow {H}_{ij}\frac{(AH)_{ij}}{{\left(H{H}^TH\right)}_{ij}}. $$

Once the similarity matrix *A* was established, the low rank solution *H* would be easily obtained. For text data, the cosine function is used to compute the similarity between two documents. For microbiome data, the Gaussian kernel function can be used to measure the similarity between different microbiome samples:

3 $$ {W}_{ij}=\exp \left(-\frac{{\left\Vert {V}_i-{V}_j\right\Vert}_F^2}{\sigma_i{\sigma}_j}\right)\left(i\ne j\right). $$

where *V*_*i*_ denotes the *i* ‐ *th* data point in original matrix. *σ*_*i*_ is the Euclidean distance between *V*_*i*_ and its *k* ‐ *th* neighbor. We set *k* to be 7 as suggested in [14]. Note that the self-similarity of the nodes is eliminated in all cases.

Next, we construct the sparse graph for microbiome sample-sample similarity network; the edge weight can be redefined as

4 $$ {W}_{ij}=\left\{\begin{array}{l}{W}_{ij}\kern1.5em \mathrm{i}\mathrm{f}\kern0.5em \mathrm{i}\in N(j)\ \mathrm{or}\kern0.5em \mathrm{j}\in N(i)\ \\ {}0\kern2.25em \mathrm{otherwise}\end{array}\right.. $$

where *N*(*i*) is the neighborhood of node *i*. In our study, we set the number of the neighbors to be 12 empirically.

Furthermore, the obtained weight matrix *W*_*ij*_ is normalized to

5 $$ A={D}^{-\raisebox{1ex}{$1$}\!\left/ \!\raisebox{-1ex}{$2$}\right.}W{D}^{-\raisebox{1ex}{$1$}\!\left/ \!\raisebox{-1ex}{$2$}\right.}. $$

where *D* is the diagonal matrix and ${D}_{ii}={\sum}_{j=1}^n{W}_{ij}$.

### Multi-view symmetric nonnegative matrix factorization

Given multi-view dataset $\left\{{V}^1,{V}^2,\cdots, {V}^{n_v}\right\}$,the corresponding similarity matrices are represented as $\left\{{A}^1,{A}^2,\cdots, {A}^{n_v}\right\}$, where *n*_*v*_ denotes the number of views. Inspired by the study [6], Multi-view symmetric nonnegative matrix factorization (Multi-view SNMF) can be formulated as

6 $$ {\displaystyle \begin{array}{l}O=\mathit{\operatorname{Min}}\left(\sum \limits_{v=1}^{n_v}{\left\Vert {A}^v-{H}^v{\left({H}^v\right)}^T\right\Vert}_F^2+\sum \limits_{v=1}^{n_v}{\gamma}^v{\left\Vert {H}^v{Q}^v-{H}^{\ast}\right\Vert}_F^2\right)\\ {}\mathrm{s}.\mathrm{t}.{H}^v,{H}^{\ast}\ge 0.\end{array}} $$

where *H*^∗^ denotes the consensus matrix toward that the solutions of all views. ${Q}^v= Diag\left(1/\sum \limits_{i=1}^m{H}_{i,1}^v,1/\sum \limits_{i=1}^m{H}_{i,2}^v,\cdots, 1/\sum \limits_{i=1}^m{H}_{i,k}^v\right)$ is an auxiliary matrix which guarantees that the clustering solution of each view is comparable. *γ*^*v*^ is the weight of the *v* ‐ *th* view and simultaneously keeps a balance between the SNMF reconstruction error and regularization term (the second term of Eq. 6). In the study, we set *γ*^*v*^ s to be equal for all views considering the convenience of computation.

Multi-view SNMF follows the basic hypothesis that there exists an underlying consensus structure in all views. This is reasonable because each view describes partial truth of the unknown; however, these limited cognitions are essential components toward objective truth.

### Hessian regularization

Given a smooth manifold *M* ⊂ *R*^*n*^, at each point *p* the tangent space is defined as *T*_*p*_(*M*) ⊂ *R*^*n*^ ⋅ *N*_*p*_ denotes the neighborhood of *p*. For each point *p*^'^ ∈ *N*_*p*_, there is a unique closest point *v*^'^ ∈ *T*_*p*_(*M*) such that the implied mapping *p*^'^ → *v*^'^ is smooth. In order to obtain the Hessian of function *f* : *M* ↦ *R*, an orthogonal coordinate system of *T*_*p*_(*M*) is needed to define. This can be achieved by the *d* largest eigenvectors of *N*_*p*_ corresponding to the orthogonal basis of *T*_*p*_(*M*). Hence, in the tangent space *f*(*p*) can be represented as *g*(*x*) : *T*_*p*_(*M*) ↦ *R*. In this way, the Hessian of *f* at point *p* can be defined as

7 $$ {\left({H}_f^{\mathrm{tan}}(p)\right)}_{i,j}=\frac{\partial }{\partial {x}_i}\frac{\partial }{\partial {x}_j}{\left.g(x)\right|}_{x=0}. $$

The previous studies point that the Frobenius form of Hessian matrix is invariant to coordinate changes [10]. Hence, the total Hessian is obtained to measure the average curviness of *f* along the manifold *M* as follows

8 $$ H(f)={\int}_{p\in M}{\left\Vert {H}_f^{\mathrm{tan}}(p)\right\Vert}_F^2 dp. $$

Hessian regularization (HR) steers the solution varying smoothly along the manifold. Compared with Laplacian regularization, Hessian fits the data perfectly and owns stronger extrapolating capability to unseen data [15]. Next, we summarize the computation process of Hessian as follows.

1. For each sample *v*_*i*_, finding its *k* nearest neighbors *N*_*i*_ and then construct the neighborhood matrix *V*^*i*^ with rows consisting of the centralized samples *v*_*j*_ = *v*_*j*_ − *v*_*i*_ for each *v*_*j*_ ∈ *N*_*i*_.
2. Conducting SVD on *V*^*i*^ so that *V*^*i*^ = *UDS*^*T*^. The first *d* columns of *U* gives the tangent coordinates of points in *N*_*i*_.
3. Constructing the matrix *M*^*i*^ = [1, *U*_.1_, *U*_.2_, ⋯, *U*_.*d*_, *U*_11_, *U*_11_, ⋯, *U*_*dd*_], where 1 denotes one vector, followed by the first *d* columns of *U* and *d* × (*d* + 1)/2 columns consisting of various cross products and squares of these *d* columns. Then, performing the Gram-Schmidt process on *M*^*i*^ and yielding $\hat{M^i}$. The last *d* × (*d* + 1)/2 columns of $\hat{M^i}$ are extracted to form *B*^*i*^ ⋅ *B*^*i*^ is the hessian matrix of the tangent space formed by the *k* nearest neighbors of the *i-th* sample.
4. Thus, a symmetric Hessian matrix can be obtained by summing up all point’s Hessian energy:

9 $$ {B}_{ij}=\sum \limits_l\sum \limits_r\left({\left({B}^l\right)}_{ri}{\left({B}^l\right)}_{rj}\right). $$

where *l* is the data point on the manifold, *i* denotes the *i* ‐ *th* data point in *N*_*l*_.

In contrast to Laplacian regularization (LR), HR can make full use of the intrinsic geometric information of the data manifold. It can not only well fit the training data, but also predict the unseen data points [16]. In this paper, we use multiple Hessian matrices obtained from different data presentations to well maintain the structural consistence in process of dimension reduction, just like with Laplacian.

### Multi-view hessian regularization based symmetric nonnegative matrix factorization

According to the analyses above, we propose a novel data integrating method, called Multi-view Hessian based symmetric nonnegative matrix factorization (MHSNMF). MHSNMF combines the advantages of SNMF and Hessian regularization, and can take full advantage of the local geometric structure information of the original data. Hence, MHSNMF theoretically owns more preferable performance.

The objective function of MHSNMF can be formulated as

10 $$ {\displaystyle \begin{array}{l}O=\mathit{\operatorname{Min}}\left\{\sum \limits_{v=1}^{n_v}{\left\Vert {A}^v-{H}^v{\left({H}^v\right)}^T\right\Vert}_F^2+\sum \limits_{v=1}^{n_v}\ {\gamma}^v{\left\Vert {H}^v{Q}^v-{H}^{\ast}\right\Vert}_F^2+\beta\ tr\left({\left({H}^{\ast}\right)}^T\left(\sum \limits_{v=1}^{n_v}\ {\alpha}^v{B}^v\right){H}^{\ast}\right)\right\}\\ {}\mathrm{s}.\mathrm{t}.{H}^v,{H}^{\ast}\ge 0,{\alpha}^v\ge 0,\sum \limits_v{\alpha}^v=1.\end{array}} $$

where *B*^*v*^ denotes the Hessian matrix derived from the *v* ‐ *th* view, *tr*(·) denotes the trace of matrix. *α*^*v*^ is the coefficient of *B*^*v*^, *β* is the regularization parameter and is used to tune the smooth of solution.

The optimal problem of MHSNMF contains three steps: (1) updating *H*^*v*^ given fixed consensus matrix *H*^∗^ and graph coefficient *α*^*v*^; (2) updating *H*^∗^ given fixed *H*^*v*^ and graph coefficient *α*^*v*^; (3) finding the optimal graph coefficients *α*^*v*^ s given fixed *H*^*v*^ and *H*^∗^. The optimizations of these three sub-problems are presented below.

1. Fixing *H*^∗^ and *α*^*v*^, computing *H*^*v*^

Given fixed *H*^∗^ and *α*^*v*^, only considering terms that are relevant to *H*^*v*^ at this step, the Eq. 10 can be reduced to

11 $$ {\displaystyle \begin{array}{l}O=\mathit{\operatorname{Min}}\left\{{\left\Vert {A}^v-{H}^v{\left({H}^v\right)}^T\right\Vert}_F^2+{\gamma}^v{\left\Vert {H}^v{Q}^v-{H}^{\ast}\right\Vert}_F^2\right\}\\ {}\mathrm{s}.\mathrm{t}.{H}^v,{H}^{\ast}\ge 0.\end{array}} $$

To minimize Eq. 11, we can solve the optimal problem with Lagrange method [6, 17]. Introducing the Lagrange multiplier *ψ*, Lagrange function can be written as

12 $$ {\displaystyle \begin{array}{l}L={\left\Vert A-H{H}^T\right\Vert}_F^2+\gamma {\left\Vert HQ-{H}^{\ast}\right\Vert}_F^2+ tr\left(\psi {H}^T\right)\\ {}\kern0.5em \propto tr\left(-2 AH{H}^T+H{H}^TH{H}^T\right)+\gamma tr\left( HQ{Q}^T{H}^T-2 HQ{H^{\ast}}^T\right)+ tr\left(\psi {H}^T\right).\end{array}} $$

For simplicity *A*, *H*, *Q* is substituted for *A*^*v*^, *H*^*v*^, *Q*^*v*^, respectively.

Taking the partial derivative of *L* with respect to *H* gives

13 $$ \frac{\partial L}{\partial H}=-4 AH+4H{H}^{\hbox{'}}H+2\gamma H Q{Q}^{\hbox{'}}-2{\gamma H}^{\ast }{Q}^{\hbox{'}}+\psi . $$

Using KKT condition, we can obtain the following updating rule

14 $$ {H}_{i,k}\leftarrow {H}_{i,k}\frac{2{(AH)}_{i,k}+\gamma {\left({H}^{\ast }{Q}^T\right)}_{i,k}}{2{\left({HH}^TH\right)}_{i,k}+\gamma {\left( HQ{Q}^T\right)}_{i,k}}. $$

(2) Fixing *H*^*v*^ and *α*^*v*^, updating *H*^∗^

This sub-problem is similar to (1), the objective function can be rewritten as

15 $$ {\displaystyle \begin{array}{l}O=\sum \limits_{v=1}^{n_v}{\gamma}^v{\left\Vert {H}^v{Q}^v-{H}^{\ast}\right\Vert}_F^2+\beta tr\left({\left({H}^{\ast}\right)}^T{BH}^{\ast}\right)+ tr\left(\psi {\left({H}^{\ast}\right)}^T\right)\\ {}\kern0.75em \propto \sum \limits_{v=1}^{n_v}{\gamma}^v tr\left(-2{H}^v{Q}^v{\left({H}^{\ast}\right)}^T+{\left({H}^{\ast}\right)}^T{H}^{\ast}\right)+\beta tr\left({\left({H}^{\ast}\right)}^T{BH}^{\ast}\right)+ tr\left(\psi {\left({H}^{\ast}\right)}^T\right).\end{array}} $$

where $$ B=\sum \limits_{v=1}^{n_v}{\alpha}^v{B}^v $$, $$ {\alpha}^v>0,\sum \limits_v{\alpha}^v=1 $$.

The rule of iteration for *H*^∗^ is given

16 $$ \kern3em {H^{\ast}}_{ij}={H^{\ast}}_{ij}\frac{{\left({\sum}_{v=1}^{n_v}{\gamma}^v{H}^v{Q}^v+\beta {B}^{-}{H}^{\ast}\right)}_{ij}}{{\left({\sum}_{i=1}^{n_v}{\gamma}^v{H}^{\ast }+\beta {B}^{+}{H}^{\ast}\right)}_{ij}}. $$

where *B* = *B*^+^ − *B*^−^. It shouldn’t be difficult to see that *H*^∗^ remains nonnegative after each iteration.

(3) Fixing *H*^*v*^ and *H*^∗^, learning *α*^*v*^

This sub-problem can be formulated as

17 $$ {\displaystyle \begin{array}{l}\min tr\left({\left({H}^{\ast}\right)}^T\left(\sum \limits_{v=1}^{n_v}\ {\alpha}^v{B}^v\right){H}^{\ast}\right).\\ {}\mathrm{s}.\mathrm{t}.{\alpha}^v\ge 0,\sum \limits_v{\alpha}^v=1\end{array}} $$

When *tr*((*H*^∗^)^*T*^*B*^*i*^*H*^∗^) the minimum one among distinct views, the solution w.r.t *α* is *α*^*i*^ = 1 and *α*^*j*^ = 0 corresponding to other views. It means that only one view takes effect and the complement information carried by multiple views cannot be utilized effectively.

In this study, we employ a trick [18, 19] to avoid this problem. We substitute (*α*^*v*^)^*r*^ for *α*^*v*^, *r* > 1. In this case, each graph has a particular contribution to the consensus matrix. The Eq. 17 can be rewritten as

18 $$ {\displaystyle \begin{array}{l}\min tr\left({\left({H}^{\ast}\right)}^T\left(\sum \limits_{v=1}^{n_v}\ {\left({\alpha}^v\right)}^r{B}^v\right){H}^{\ast}\right).\\ {}\mathrm{s}.\mathrm{t}.{\alpha}^v\ge 0,\sum \limits_v{\alpha}^v=1\end{array}} $$

To solve Eq. 18, we introduce Lagrange multiplier *λ* and consider the constraint $\sum \limits_v{\alpha}^v=1$ and then obtain the Lagrange function

19 $$ L\left(\alpha, \lambda \right)= tr\left({\left({H}^{\ast}\right)}^T\left(\sum \limits_{v=1}^{n_v}\ {\left({\alpha}^v\right)}^r{B}^v\right){H}^{\ast}\right)-\lambda \left(\sum \limits_{v=1}^{n_v}{\alpha}^v-1\right). $$

Taking the partial derivative of *L*(*α*, *λ*) with respect to *α*^*v*^ and *λ* set them to zero

20 $$ \left\{\begin{array}{l}\frac{\partial L}{\partial {\alpha}^v}=r{\left({\alpha}^v\right)}^{r-1} tr\left({\left({H}^{\ast}\right)}^T{B}^v{H}^{\ast}\right)-\lambda =0,\kern1em v=1,2,\cdots, {n}_v\\ {}\frac{\partial L}{\partial \lambda }=\sum \limits_{v=1}^{n_v}{\alpha}^v-1=0\end{array}\right.. $$

Finally, a closed solution of *α*^*v*^ can be given

21 $$ {\alpha}^v=\frac{{\left(1/ tr\left({\left({H}^{\ast}\right)}^T{B}^v{H}^{\ast}\right)\right)}^{1/r-1}}{\sum \limits_{v=1}^{n_v}{\left(1/ tr\left({\left({H}^{\ast}\right)}^T{B}^v{H}^{\ast}\right)\right)}^{1/r-1}}. $$

From Eq. 21 we can see that *α*^*v*^ is always nonnegative because Hessian matrix *B*^*v*^ is SDP.

Table 1 gives the pseudocode of the proposed MHSNMF.

**Table 1:** The pseudocode of MHSNMF

MHSNMF algorithm	
Input: $\left\{{V}^1,{V}^2,\cdots, {V}^{n_v}\right\}$, *γ*^*v*^_,_ *α*^*v*^_,_ *k*	
Output: $\left\{{H}^1,{H}^2,\cdots, {H}^{n_v}\right\}$, *H*^∗^_,_ *α*^*v*^	
1. Transforming each *V*^*i*^ to *A*^*i*^ according to Eqs.3, 4 and 5	
2. Solving the Hessian matrix *B*^*i*^ for each view *V*^*i*^	
3. Initializing *H*^*i*^_,_ *H*^∗^_,_ *α*^*v*^ = 1/*n*_*v*_	
4. Iteration beginning	
For *i* = 1 : *n*_*v*_,	
Fixing *H*^∗^_,_ *α*^*v*^_,_ updating *H*^*v*^ according to Eq. 14	
Fixing *H*^*v*^_,_ *α*^*v*^_,_ updating *H*^∗^ according to Eq. 16	
Learning *α*^*v*^ according to Eq. 21	
Until all views have been updated	
5. Repeating	

### Datasets and evaluation metrics

#### Datasets

In this paper, two public multi-view datasets are used to verify the performance of the proposed MHSNMF algorithm.

1. Three-source text story dataset. The dataset was collected from three online news sources: BBC, Reuters and the Guardian. One hundred sixty-nine stories were reported in all three sources. Each of them was manually classified into one of the six topical labels: business, entertainment, politics, sport, health and technology. These roughly correspond to the principal section headings used across these three sources. To facilitate comparisons using the AC and NMI metrics, only the main topic for each story was considered. More details can be found in [20]. Table 2 describes the detailed statistical information.
2. Human microbiome dataset (HMP). This dataset includes three compositional profiles: phylogenetic, metabolic and transporter profiles from HMP site. It consists of 637 samples drawn from seven body sites including one vagina (posterior fornix), one gut (stool), one nasal (anterior nares), one skin (retroauricular crease), and three oral sties (supragingvial plaque, tongue dorsum and buccal mucosa). The phylogenetic profile which contains the microorganism relative abundances was estimated by software MetaPhlAn at species level (710 × 637). For functional profile, the transporter profile (4941 × 637) and the metabolic profile (295 × 637) are investigated by filtering out those with low variances (see Table 3 for the detailed statistical summary) [4]. All the data can be available from HMP site: http://hmpdacc.org/ [21].

**Table 2:** Statistics of the Three-source dataset

Topics	# Samples
Business	56
Entertainment	21
Health	11
Politics	18
Sport	51
Technology	12

**Table 3:** Statistics of the HMP dataset

Body sites	# Samples
Stool	134
Posterior_fornix	49
Anterior_nares	86
Buccal_mucosa	106
Plaque	122
Retroauricular_crease	17
Tongue_dorsum	123

#### Evaluation metrics

In the following experiments, two frequently used metrics are applied to evaluate the clustering performance of MHSNMF, i.e. accuracy (AC) and normalized mutual information (NMI). Generally speaking, higher AC or NMI indicates the better clustering performance. More details were described in [22].

## Results and discussion

### Experimental results

In this section, we conduct extensive experiments to elucidate the effectiveness of the proposed MHSNMF approach. Some baseline algorithms below are compared:

- Single view (BSSV and WSSV). Running standard SNMF on each view, BSSV is the most informative view that has the best clustering quality; WSSV refers to the worst view.
- Multi-NMF. Iteratively fusing the coefficient matrices learnt from different views to form a consensus clustering solution. In the fusion process, coefficient matrix from each view is normalized to guarantee that they are comparable and meaning [6].
- Co-training spectral clustering (Co-training SC). Performing multi-view spectral clustering with co-training paradigm [23] to update iteratively the graph structure of one view by using the discriminative eigenvectors obtained from the other view.
- Similarity network fusion (SNF). Constructing similarity network for each view and then iteratively fusing these networks so that global and local information from different views can be shared and interchanged. More details can be obtained from [24].
- LJ-NMF. Fixing a common coefficient matrix across different views and then performing joint nonnegative matrix factorization as shown in [4].
- CSMF. Extracting common and specific patterns from multiple data generated under interrelated biological scenarios via nonnegative matrix factorization [7].
- NetNMF. Utilizing Tri-factor NMF to construct two layer modular networks. For each biological network, the samples were reordered according to the obtained features modules. At last, the optimal clustering performance is recorded [9].
- MHSNMF. This is the proposed algorithm. In the experiments, we used NNDSVD method to enhance the initiation stage of MHSNMF [25]. The parameter selection will be discussed later.

Table 4 shows the clustering results of different algorithms on these two datasets. From this table, we can see that MHSNMF outperforms the baseline and the state-of-art algorithms in terms of AC and NMI.

**Table 4:** The best clustering performance on two datasets

	Accuracy (%)		NMI (%)	
	Three-source	HMP	Three-source	HMP
BSSV	79.88	88.54	69.66	84.64
WSSV	65.68	81.16	58.26	80.71
Multi-NMF	66.86	77.55	55.04	72.87
Co-training SC	61.54	63.58	58.03	63.68
SNF	65.68	91.21	56.34	89.20
LJ-NMF	69.82	73.16	60.08	67.77
CSMF	65.18	74.01	63.23	65.43
NetNMF	70.18	82.50	61.24	81.76
MHSNMF	82.84	95.28	71.43	91.76

In Multi-NMF, these clustering results on three-source and HMP data are obtained when *γ*^*v*^ = 0.01 and 0.05, respectively. For three-source dataset, the cosine function was used to construct the similarity matrix. For BSSV, WSSV and LJ-NMF, the number of neighborhoods on HMP data was set to be 12. For other values, MHSNMF still outperforms other algorithms in most cases.

As we can see, on these two realistic dataset MHSNMF achieves much improvement in terms of AC and NMI compared with other algorithms. One of the possible reasons is that MHSNMF takes advantage of the local geometry information reserved in the data to satisfy the manifold consistency assumption well. The proposed MHSNMF algorithm can effectively find the latent consensus clustering solution across different views.

### Parameter tuning

There are two types of parameters in the proposed MSNMF algorithm: *γ*^*v*^ and *β*. *γ*^*v*^ is the regularization parameter for the *v* ‐ *th* view. On one hand, *γ*^*v*^ reflects each view’s relative importance among all views, on the other hand, it also indicates the strength which we want to impose on the regularization constraint. Considering the convenience of computation, we set *γ*^*v*^ s to be equal for each view. *β* is the graph regularization parameter. In our experiment the values of *β* are tuned from the candidate set {10^−4^, 5 × 10^−4^, 10^−3^, 5 × 10^−3^, 10^−2^, 0.05, 0.1, 0.5, 1} and *γ*^*v*^ is set to vary in the set {10^−3^, 5 × 10^−3^, 10^−2^, 0.05, 0.1, 0.5, 1} for all the datasets. Besides, in computing Hessian the size of neighborhood is set to be 30.

**Fig. 2:**
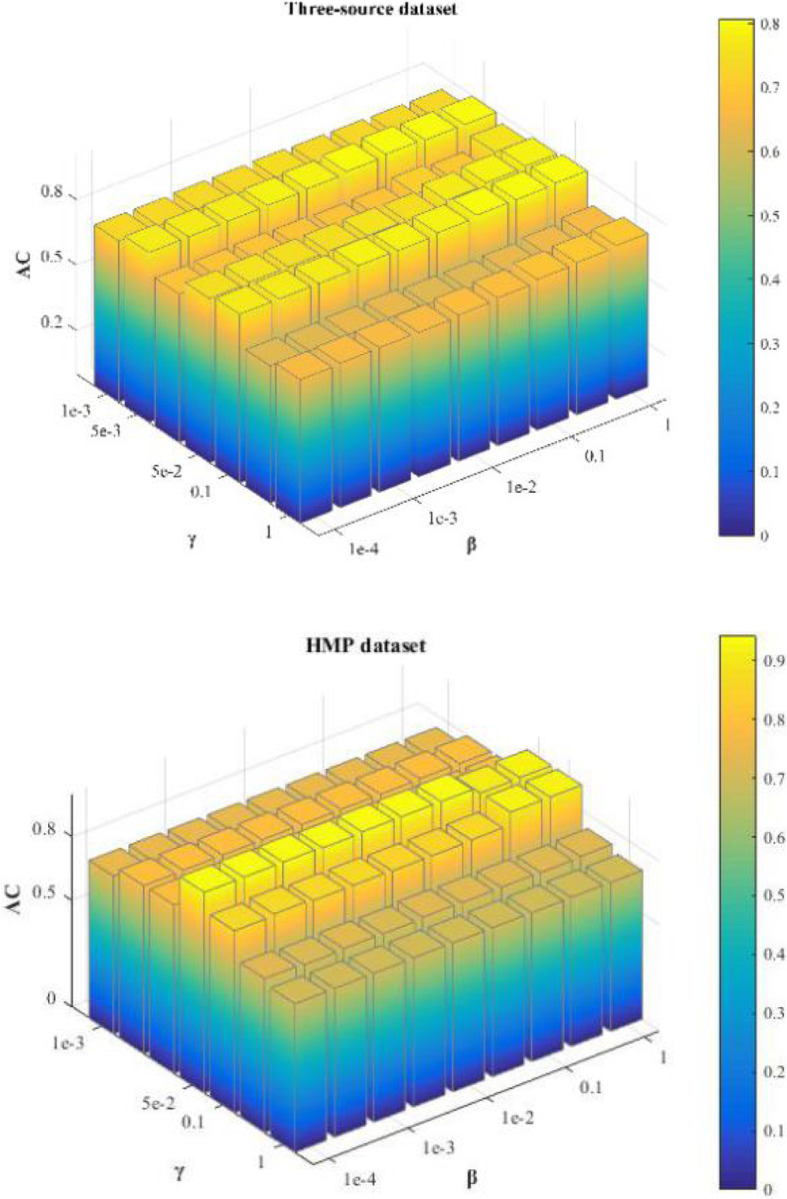
The performance of MHSNMF w.r.t parameters *γ* and *β* on three-source and HMP datasets, respectively

Figure 2 shows how the performance of MHSNMF varies with changes of parameters *γ*^*v*^ and *β* on these two datasets. As Fig. 2 shown, MHSNMF obtains the best performance when *γ* equals to 0.1 and *β* equals to 0.5 on three-source data. Moreover, for other values of *β* MHSNMF still owns stable and reliable performance. On HMP dataset, MHSNMF performs relatively stable when *γ* equals to 0.05 and *β* varies during the set {10^−4^, 5 × 10^−4^, 10^−3^, 5 × 10^−3^, 10^−2^, 0.05, 0.1}.

### Convergence curve and the performance

**Fig. 3:**
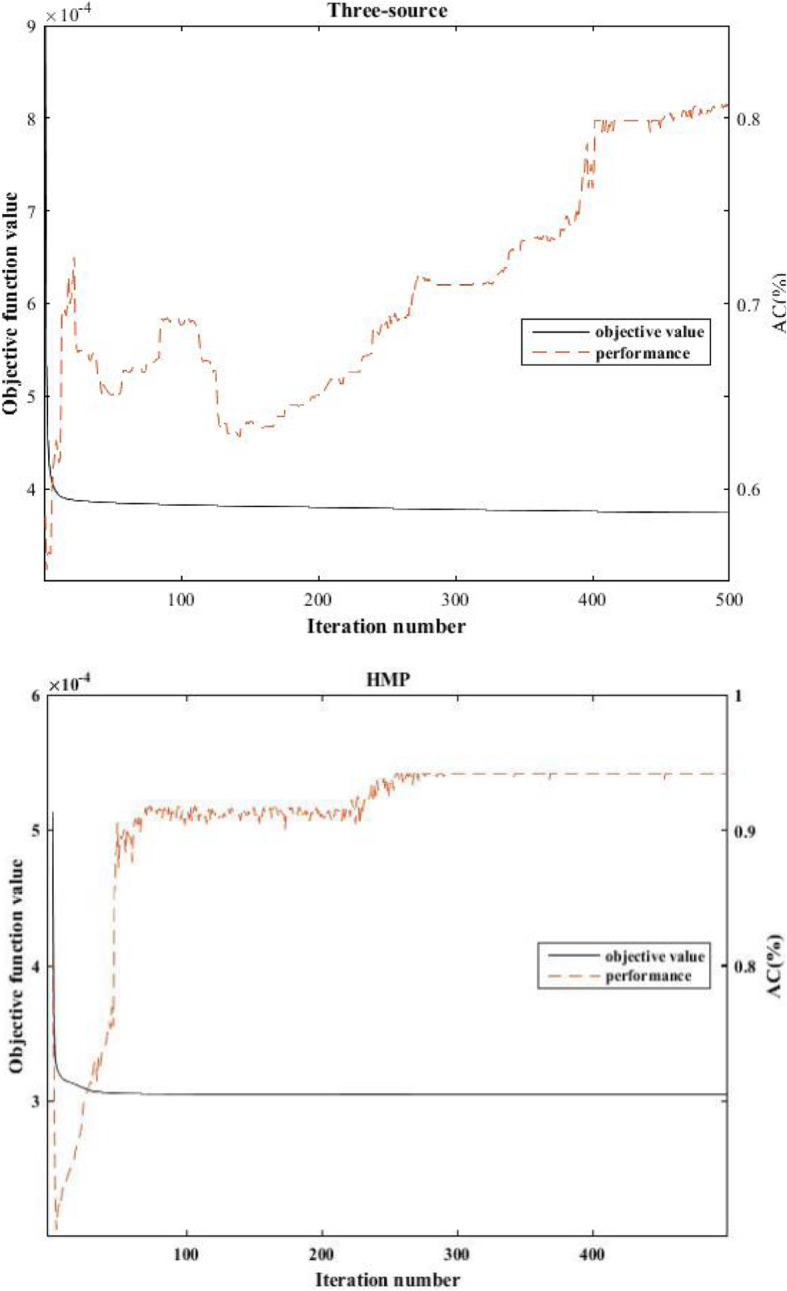
Convergence and corresponding AC curve of MHSNMF on three-source and HMP datasets

According to the iterative rules (Eqs. 14, 16 and 21), the objective function value progressively grows smaller and it is convergent. Figure 3 shows the convergence curves along with the accuracy value on these two datasets, respectively. The results below are obtained when *γ* is set to be 0.05 and *β* is set to 0.01. As we can see that MHSNMF will converge after a few iterations. Interestingly, on three-source data the performance curve shows some shocks in the iterative process. One of the possible reasons is that the clustering solutions obtained from multiple views may not be misaligned for each cluster. This is beyond the scope of this paper.

As Fig. 3 shown, on HMP dataset the performance of MHSNMF achieves the optimal value 95.28%/91.76% in terms of AC/NMI after around 250 iterations. It is worth noting that MHSNMF converges very fast regardless of Three-source or HMP data. This suggests the effectiveness and efficiency of MHSNMF for clustering multi-view omics data.

### Parameter study

**Fig. 4:**
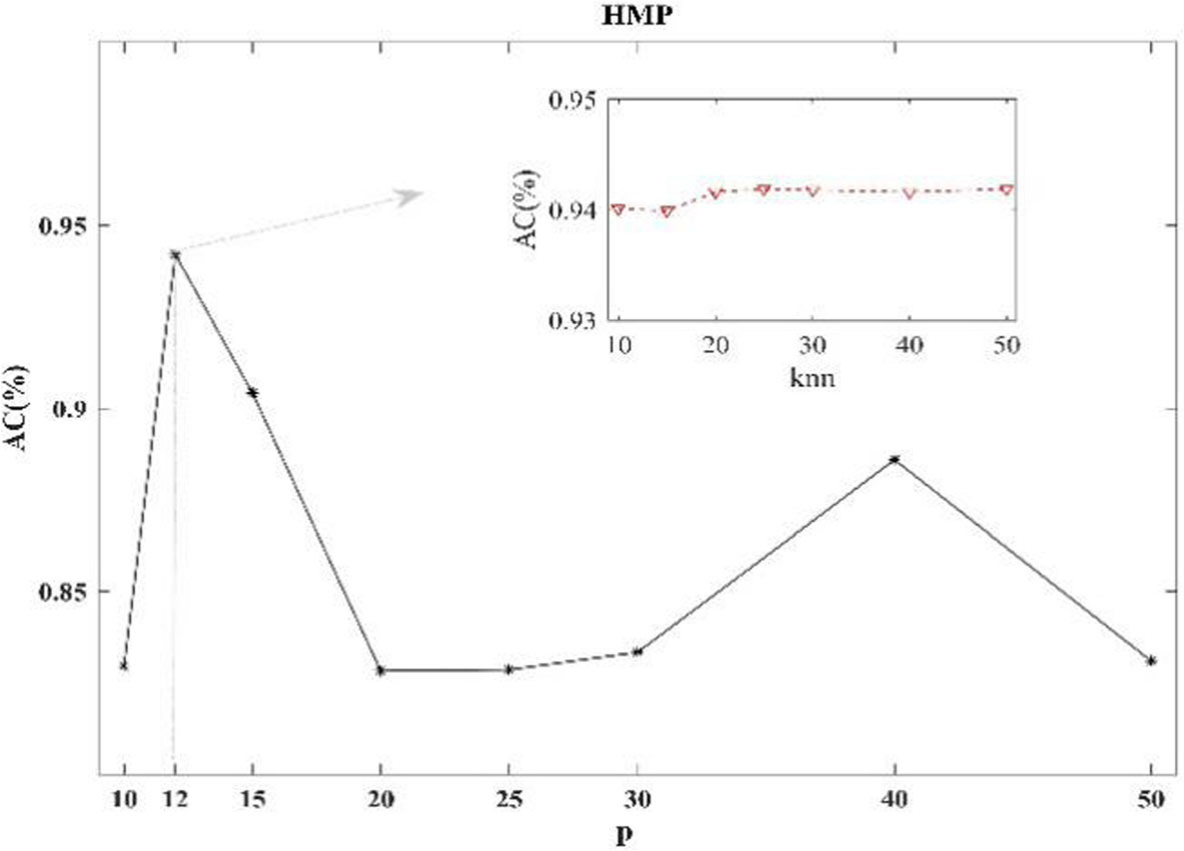
Performance of MHSNMF versus *p* and *knn* on HMP data

In this subsection, extensive experiments are conducted on HMP data to further validate the performance of MHSNMF w.r.t the number of neighbors *p* and *knn* in computing Hessian and constructing affinity graphs, respectively. Figure 4 demonstrates how the accuracy varies with changes in the number of neighbors.

As Fig. 4 shown, the accuracy of MHSNMF achieves the best value when *p* is set to be 12. Meanwhile, the performance of MHSNMF is stable for the various values of *knn*. For other values of *p*, in most cases AC doesn’t vary significantly with the changes of *knn*, which demonstrates the number of neighbors in computing Hessian cannot have a remarkable impact on the performance of MHSNMF on HMP dataset. This is important to study the microbiome data. We can set a fixed *knn* value in computing Hessian for the convenience of computation. This study also offers a new reference for multiple heterogeneous omics data fusion.

### Analysis on HMP data

**Fig. 5:**
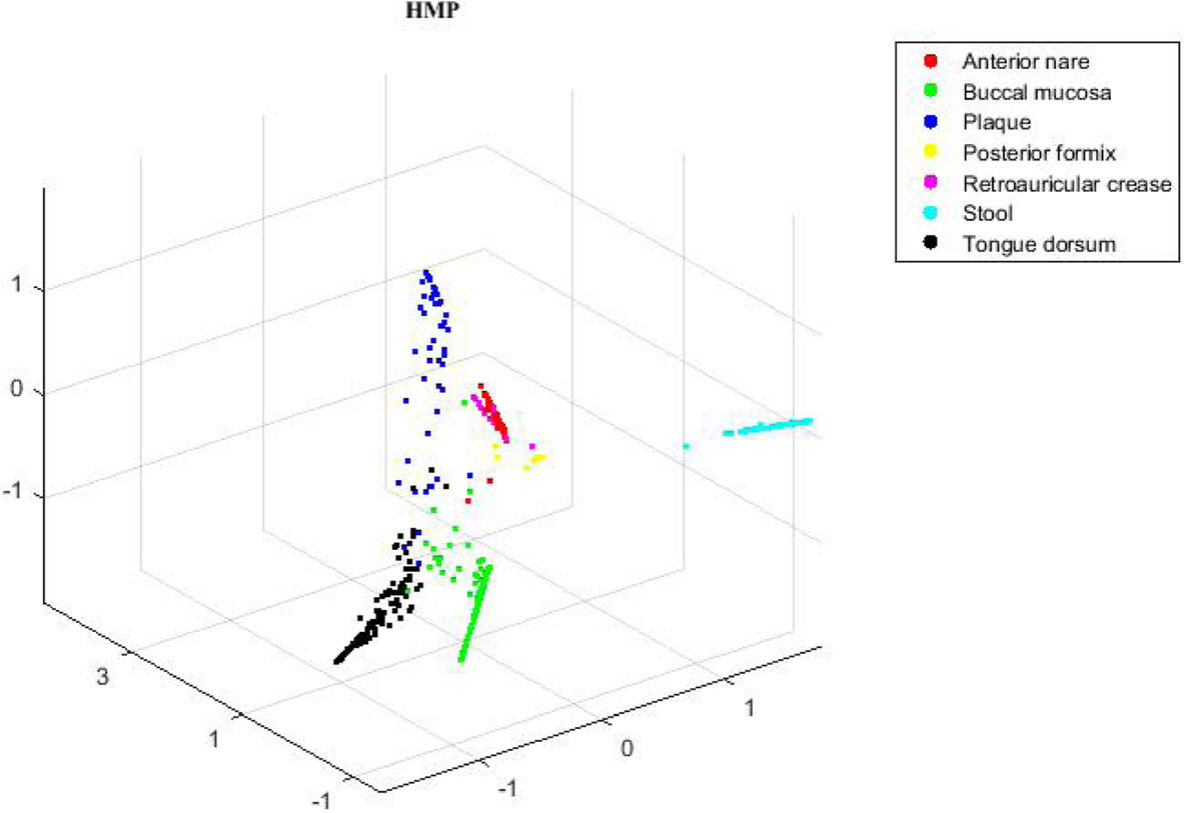
Scatter plot of HMP data in three-dimension space. The result is obtained when *γ* equals to 0.05 and is set to be 1e-4. Seven colors indicate the true labels of microbiome samples from different body sites

To further explore the structures and functions of human microbiome, we apply the proposed MHSNMF algorithm to HMP data and find that it is very useful. Classical multidimensional scaling (MDS) is used on the consensus matrix *H*^∗^ to describe the relationships among microbiome samples in three dimensional space. Figure 5 reveals clear clustering patterns derived from the consensus matrix. This supports Jeffery et al.‘s argument that the change at the species level of human microbiome is irrelevant to the discrete clusters (enterotype), but it is continuous [26].

As Fig. 5 shown, MHSNMF clearly identifies different clusters corresponding to microbiome samples from seven different body sites. Theses samples from anterior nares (red), gut (cyan) and posterior fornix (yellow) are well separated, particularly for gut microbiome samples. One possible reason is that gut microbiome has more complicated composition and spatial distance relative to other sites. We can also find that samples from three oral sites (buccal mucosa, plaque, tongue dorsum) may have overlapped with each other. This might be because these three sites are all from oral cavity. Therefore, theses samples may have similar microbiome composition and diversity.

### Other application

Besides clustering, MHSNMF has also other potential application, for instance, predicting the classification of new samples via consensus matrix *H*^∗^ obtained from multiple views. When applied it to HMP data with multiple views, the Eq. (10) can also be understood as finding a consensus basis *H*^∗^ (similar to basis matrix in NMF), such that in the space spanned by *H*^∗^ the presentation of new microbiome samples can also reflect their structure information. Therefore, we can express a new microbiome sample *x*_*new*_ as *h* by solving the following optimization problem:

22 $$ \underset{h\ge 0}{\min }{\left\Vert S-{H}^{\ast }h\right\Vert}_F^2+\alpha {\left\Vert h\right\Vert}_2^2. $$

Where, $S={V}_{tr}^i\ast {x}_{new}$, ${V}_{tr}^i$ is training set from the view, the second term is L_2_ regularization term.

We can use closeness of *h* to the rows of *H*^∗^ to decide how likely the new microbiome sample should belong to which body site. For example, one can predict the class of a new microbiome sample according to *knn* method.

To evaluate our approach, we recollect and extend human microbiome samples to 653 cases, and then separate HMP data (phylogenetic profile and metabolic profile) into training set and test set by randomly selecting 70% samples from each body site as training set and the remaining samples as test set. We firstly learn a consensus matrix *H*^∗^ from phylogenetic profile and metabolic profile samples in training set, and then predict the classification of phylogenetic (or metabolic) samples in the test set.

To verify that the consensus *H*^∗^ computed by the proposed MHSNMF algorithm indeed well represents the geometric structure, we also compare several baseline approaches. One is to learn the matrix *H*^*i*^ only by single view SNMF, the remaining steps for making predictions are the same as MHSNMF. The other two methods based on subspace learning are Canonical Correlation Analysis (CCA) and Partial Least Squares Regression (PLSR) [27]. We use the consensus matrix *H*^∗^ to predict the classification of new samples from each view. The experimental results are shown in Table 5.

**Table 5:** The prediction accuracy on HMP data

	Phylogenetic profile (%)	Metabolic profile (%)	Average (%)
SNMF	91.08%	90.71%	90.90%
CCA	64.58%	21.88%	43.23%
PLSR	91.67%	69.27%	80.47%
MHSNMF	**94.27%**	**93.83%**	**94.06%**

As Table 5 shown, MHSNMF obtains much improvement in accuracy compared with three baselines methods on HMP data. It should be noted that CCA fails to utilize the complementary information from multiple views and cannot find the underlying subspace shared by multiple biological compositional profiles. One possible reason is that the objective of CCA is to find the maximum linear correlation between two feature profiles data. Therefore, CCA-based methods may be not suitable for data with nonlinear structure, such as microbiome data. In contrast, by adopting graph and Hessian regularization framework to learn the consensus matrix *H*^∗^ across all views, MHSNMF succeeds in capturing such knowledge.

## Conclusions

In this paper, we introduced a novel multi-view Hessian regularization based symmetric nonnegative matrix factorization algorithm (MHSNMF) for multiple omics data integration task. On human microbiome data, the proposed MHSNMF algorithm can effectively combine the phylogenetic, transporter, and metabolic profiles into a unified paradigm to analyze the relationships among different microbiome samples. Experimental results demonstrate MHSNMF has the latent application in multiple biological profiles data analysis. Furthermore, the prediction method based on MHSNMF has shown to be effective in judging the types of new microbiome samples.

To our best knowledge, the interactions among microorganisms are complicated owning to the influences from host environment, diet and other species, particularly for the intestinal flora. Dissecting and exploring the structure and functions of intestinal microbiota is an essential step toward understanding the occurrence and development of microbiota-related disease. In the future, combining the phylogenetic information of species into the microbial interaction network to analyze functional modules is our next consideration.

## Data Availability

The datasets generated or analyzed during the current study are available in the GitHub repository, https://github.com/chonghua-1983/MHSNMF.

## Abbreviations

HMP: Human Microbiome Plan
iHMP: Integrative Human Microbiome Plan
MetaHIT: Metagenomics of the Human Intestinal Gut
NMF: Nonnegative Matrix Factorization
GNMF: Graph Regularized Nonnegative Matrix Factorization
SNMF: Symmetric Nonnegative Matrix Factorization
MHSNMF: Multi-view Hessian Regularization based Symmetric Nonnegative Matrix Factorization
SVD: Singular Value Decomposition
AC: Accuracy
NMI: Normalized Mutual Information
BSSV: Best Single view
WSSV: Worst Single View
Multi-NMF: Multi-view Nonnegative Matrix Factorization
Co-training SC: Co-training spectral clustering
SNF: Similarity network fusion
LJ-NMF: Joint Nonnegative Matrix Factorization with Laplacian
CSMF: Common and Specific Matrix Factorization
NetNMF: Two Layers Network based Nonnegative Matrix Factorization
